# Draft genome sequence of *Bacillus atrophaeus* SHZ-24 isolated from cotton (*Gossypium hirsutum*) in the arid region of northwest China

**DOI:** 10.1128/mra.01005-25

**Published:** 2026-02-09

**Authors:** Yun Chen, XinXiang Niu, Ablimit Nuraliya, Yue Sheng, Hongmei Yang, Min Chu, Ning Wang, Huifang Bao, Faqiang Zhan, Rong Yang, Kai Lou, Shuang Dou, Zhao Zhang, Zhichao Meng, Yingwu Shi, Deying Ma

**Affiliations:** 1 College of Agronomy, Xinjiang Agricultural University 117840, Urumqi, Xinjiang, China; 2 Institute of Microbiology, Xinjiang Academy of Agricultural Sciences 655642 , Urumqi, Xinjiang, China; 3 Xinjiang Laboratory of Special Environmental Microbiology, Urumqi, Xinjiang, China; 4 Institute of Agricultural Resources and Environment, Xinjiang Academy of Agricultural Sciences, Urumqi, Xinjiang, China; 5 Key Laboratory of Agricultural Environment in Northwest Oasis of Ministry of Agriculture and Countryside, Urumqi, Xinjiang, China; 6 College of Life and Science and Technology, Xinjiang University 682541, Urumqi, Xinjiang, China; Wellesley College, Wellesley, Massachusetts, USA

**Keywords:** bacteria, *Bacillus atrophaeus*, gram-positive bacteria

## Abstract

In this paper, we report the draft genome sequence of *Bacillus atrophaeus* SHZ-24 isolated from cotton rhizosphere. The average GC content of *B. atrophaeus* SHZ-24 was 43.15%. The genome of *B. atrophaeus* SHZ-24 contains 4,012 protein-coding sequences.

## ANNOUNCEMENT

*Bacillus atrophaeus* is a gram-positive, aerobic, melanin-producing bacterium that can produce melanin on tyrosine medium (1). *B. atrophaeus* has played an important role in the study of spore inactivation, as a plant growth promoter and crop protectant and as a producer of different biomolecules (2–5). To gain a better understanding of *Bacillus atrophaeus* and its response mechanisms to environmental stress, we sequenced and annotated the genome of *B. atrophaeus* SHZ-24. This strain was isolated from a desert gray soil sample collected from the cotton rhizosphere—less than 0.5 cm from the root—from an agricultural field in Shihezi, Xinjiang, China (44.20° N, 86.03° E, altitude 416 m).Rhizosphere soil bacteria were isolated by dilution coating plate method. Each soil sample was serially diluted to 10^−6^ and plated onto TSA medium and incubated at 33°C for 24 h. The field efficacy test was carried out with 100 billion CFU/g *B. atrophaeus* SHZ-24 powder, and the control effect was as high as 68.76% (6). The DNAMAN 8.0 software was used to calculate the similarity value of the 16S rRNA gene sequence using primers 27F(5′-AGAGTTTGATCCTGGCTCAG-3′) and 1492R(5′-GGTTACCTTGTTACGACTT-3′) (2), which showed it was 99.31% identical to *B. atrophaeus* (AB021181) (7).

The genomic DNA was extracted using the Ezup Column Bacteria Genomic DNA Purification Kit (Sangon Biotech, China) following the manufacturer’s protocol. DNA concentration was quantified using a NanoDrop 2000 spectrophotometer (Thermo Fisher Scientific, USA), and Illumina sequencing libraries were prepared from the sheared fragments using the NEXTFLEX Rapid DNA-Seq Kit, according to the instructions provided by the manufacturer and sequenced using Illumina HiSeq 4000 in paired-end reads at the Majorbio sequencing facility. The generated raw paired-end fastq reads (2 × 150 bp) were quality checked using FastQC (v.0.11.7) (8) followed by trimming of low-quality bases using Trimmomatic (v.0.39) (9), and the data quality was rechecked using FastQC (v.0.11.7) (8). The cleaned reads were assembled using SPAdes (v.3.15.5) (10). Quast (v.5.0.2) (6) was used to evaluate the genome assembly quality. The completeness and contamination were assessed with CheckM (v.1.1.6) (11). The assembled draft genome was annotated on the Rapid Annotation System Technology Pipeline (12) and the NCBI Prokaryotic Genome Annotation Pipeline (v.6.5) (13). The annotated genomes were assessed against the Genome Taxonomy Database (GTDB) using GTDB-Tk software (v.1.7.0) (14).

**Fig 1:**
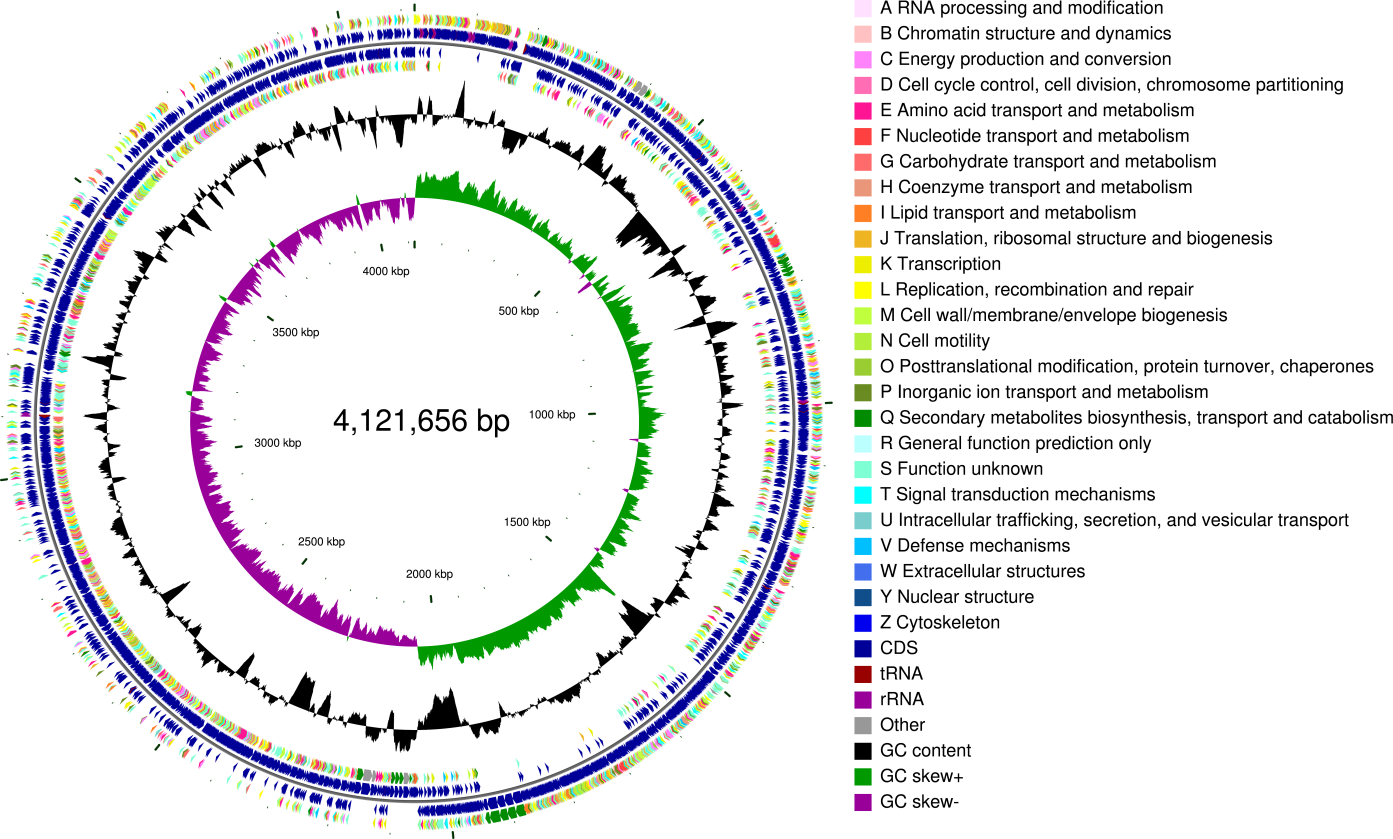
The CGView map of *Bacillus atrophaeus* SHZ-24 genome. The outermost circle of the circle graph is the identification of genome size; the second and third circles are coding sequences (CDSs) on the positive and negative chains, and different colors indicate the functional classification of different COGs of CDS; the fourth circle is the rRNA and tRNA; the fifth circle is the GC content. The outward red part indicates that the GC content in this region is higher than the average GC content of the whole genome. The higher the peak value, the greater the difference between the average GC content. The inward blue part indicates that the GC content in this region is lower than the average GC content of the whole genome. The higher the peak value is, the greater the difference between the average GC content and the average GC content is. The innermost circle is the GC-skew value, and the specific algorithm is G−C / G+C, which can assist in judging the leading chain and the lagging chain. In general, the leading chain has a GC skew >0, whereas the lagging chain has a GC skew <0.

According to the results of genome sequencing, the whole genome of *B. atrophaeus* SHZ-24 consists of a 4,267,134 bp chromosome, 458,578 reads, 4,081 protein-coding sequences, an average G+C content of 43%, 3 contigs, and 45× genome coverage. The N50 and L50 values of the assembly were 4,121,656 bp and 1, respectively. The genome map of strain SHZ-24 was generated using Circos (v.0.64) (Fig. 1). The focus is on the mining of carbohydrate-active enzyme genes and the mining of genes related to secondary metabolite synthesis gene clusters. The glycoside hydrolase putative gene and non-ribosomal peptide synthetase putative gene were detected. A total of 375 virulence putative genes and 232 drug resistance putative genes were detected.

## Data Availability

The whole-genome shotgun project for *Bacillus atrophaeus* SHZ-24 has been deposited at DDBJ/ENA/GenBank under accession number CP174157; CP174157 is composed of chromosome CP174157.1, plasmid A CP174158.1, and plasmid B CP174159.1. The raw reads are available under BioProject accession number PRJNA1149161, and the BioSample accession number is SAMN43222669. The sequence data obtained in this work have been deposited in the NCBI Sequence Read Archive under accession number SRR33830715.

## References

[1] Vos P, Garrity G, Jones D. 2009. The Firmicutes. Bergey’s manual of systematic bacteriology. Vol. 3. Springer Nakamura LK, New York.

[2] Fritze D, Pukall R. 2001. Reclassification of bioindicator strains Bacillus subtilis DSM 675 and Bacillus subtilis DSM 2277 as Bacillus atrophaeus. Int J Syst Evol Microbiol 51:35–37. doi:10.1099/00207713-51-1-35 11211269

[3] Sella S, Gouvea PM, Gomes VF, Vandenberghe LPS, Minozzo JC, Soccol CR. 2013. Glycerol-based sterilization bioindicator system from Bacillus atrophaeus: development, performance evaluation, and cost analysis. Appl Microbiol Biotechnol 97:1031–1042. doi:10.1007/s00253-012-4350-3 22911095

[4] Mohammadipanah F, Lotfi M, Eskandari N. 2016. Efficacy of nanostructures as preservation carriers of Bacillus atrophaeus in the preparation of sterilization bioindicators. J Pharm Innov 11:323–330. doi:10.1007/s12247-016-9260-y

[5] Shintani H. 2011. Validation of sterilization procedures and usage of biological indicators in the manufacture of healthcare products. Biocontrol Sci 16:85–94. doi:10.4265/bio.16.85 21946318

[6] Gurevich A, Saveliev V, Vyahhi N, Tesler G. 2013. QUAST: quality assessment tool for genome assemblies. Bioinformatics 29:1072–1075. doi:10.1093/bioinformatics/btt086 23422339 PMC3624806

[7] Yu MC, Yang CX, Wang JZ, Hou QS, Zhang S, Cao MJ. 2021. First report of tomato spotted wilt virus isolated from nasturtium (Tropaeolum majus) with a serious leaf mosaic disease in China. Plant Disease 105:716–716. doi:10.1094/PDIS-03-20-0688-PDN

[8] Andrews S. 2010. Fastqc: a quality control tool for high throughput sequence data

[9] Bolger AM, Lohse M, Usadel B. 2014. Trimmomatic: a flexible trimmer for Illumina sequence data. Bioinformatics 30:2114–2120. doi:10.1093/bioinformatics/btu170 24695404 PMC4103590

[10] Bankevich A, Nurk S, Antipov D, Gurevich AA, Dvorkin M, Kulikov AS, Lesin VM, Nikolenko SI, Pham S, Prjibelski AD, Pyshkin AV, Sirotkin AV, Vyahhi N, Tesler G, Alekseyev MA, Pevzner PA. 2012. SPAdes: a new genome assembly algorithm and its applications to single-cell sequencing. J Comput Biol 19:455–477. doi:10.1089/cmb.2012.0021 22506599 PMC3342519

[11] Parks DH, Imelfort M, Skennerton CT, Hugenholtz P, Tyson GW. 2015. CheckM: assessing the quality of microbial genomes recovered from isolates, single cells, and metagenomes. Genome Res 25:1043–1055. doi:10.1101/gr.186072.114 25977477 PMC4484387

[12] Aziz RK, Bartels D, Best AA, DeJongh M, Disz T, Edwards RA, Formsma K, Gerdes S, Glass EM, Kubal M, et al.. 2008. The RAST server: rapid annotations using subsystems technology. BMC Genomics 9:75. doi:10.1186/1471-2164-9-75 18261238 PMC2265698

[13] Tatusova T, DiCuccio M, Badretdin A, Chetvernin V, Nawrocki EP, Zaslavsky L, Lomsadze A, Pruitt KD, Borodovsky M, Ostell J. 2016. NCBI prokaryotic genome annotation pipeline. Nucleic Acids Res 44:6614–6624. doi:10.1093/nar/gkw569 27342282 PMC5001611

[14] Chaumeil P-A, Mussig AJ, Hugenholtz P, Parks DH, Hancock J. 2019. GTDB-Tk: a toolkit to classify genomes with the Genome Taxonomy Database. Bioinformatics 36:1925–1927. doi:10.1093/bioinformatics/btz848 31730192 PMC7703759

